# Impaired T-Cell Function in B-Cell Lymphoma: A Direct Consequence of Events at the Immunological Synapse?

**DOI:** 10.3389/fimmu.2015.00258

**Published:** 2015-06-02

**Authors:** Marian Nassef Kadry Naguib Roufaiel, James W. Wells, Raymond J. Steptoe

**Affiliations:** ^1^The University of Queensland Diamantina Institute, The University of Queensland, Translational Research Institute, Brisbane, QLD, Australia

**Keywords:** lymphoma, immunological synapse, T-cell tolerance, T-cell function

## Abstract

Tumors can escape immune destruction through the development of antigen loss variants and loss of antigen processing/presentation pathways, thereby rendering them invisible to T cells. Alternatively, mechanisms of peripheral T-cell tolerance that would normally be important for protection from the development of autoimmunity may also be co-opted to (i) generate an immuno-inhibitory tumor environment, (ii) promote development of regulatory cell populations, or (iii) cell-intrinsically inactivate tumor-specific T cells. Emerging evidence suggests that T-cell function is impaired in hematological malignancies, which may manifest from cognate interactions between T cells and the tumor. The immunological synapse forms the cognate T-cell and antigen-presenting cell interaction and is the site where key signalling events, including those delivered by co-inhibitory receptors, that determine the fate of T cells occur. Here, we review evidence that events at the immune synapse between T cells and malignant B cells and alterations in immune synapse function may contribute to loss of T-cell function in B-cell malignancies.

## B-Cell Lymphoma and T-Cell Responses

Lymphomas are a range of lymphoid tissue malignancies arising principally from B cells, but a minority (<10%) are derived from T cells (1). Broadly, two categories of B-cell lymphoma are recognized. Hodgkin’s lymphoma (HL), characterized by multinucleated Reed–Sternberg cells in affected sites, and non-Hodgkin’s lymphoma (NHL), which are highly diverse malignancies constituting up to 90% of lymphoma cases in developed countries. It was estimated that in the United States, lymphoma would represent approximately 5% of newly diagnosed cancers and account for approximately 18,990 deaths in 2014 (2, 3). In B-cell chronic lymphocytic leukemia (B-CLL), which is the most common chronic leukemia, malignant B cells accumulate in blood and bone marrow. While classified as different diseases, similar treatment challenges exist for B-cell lymphoma and B-CLL.

## Modulation of T-Cell Responses by B-Cell Malignancies

A key risk factor for development of B-cell lymphoma is immunodeficiency or immune suppression (4). Patients with HIV-1 and AIDS and children with primary immunodeficiency diseases have elevated rates of B-lymphoma, and an aggressive form of EBV^+ve^ lymphoma, post-transplant lymphoproliferative disease (PTLD), is common in immunosuppressed transplant recipients (5–7). These observations strongly suggest that effective T-cell responses are required for prevention and control of B-cell malignancies. Emergence of antigen loss variants and disruption of antigen processing/presentation pathways can contribute to immune escape of B-lymphomas (8–11). In addition, metabolically hostile and immune-suppressive tumor microenvironments (12–15), and/or expansion or induction of immunosuppressive cells, such as regulatory T cells (16), myeloid-derived suppressor cells, and immunosuppressive macrophages (17), may also play a role. However, compelling evidence suggests that B-cell malignancies induce T-cell intrinsic alterations resulting in loss of T-cell function. In EBV^+ve^ lymphoma, T-cell responses to EBV proteins serve as surrogates for tumor-specific responses and are likely indicators of the response of T-cells to non-viral lymphoma antigens. CD8^+^ T-cell responses to latency-phase proteins expressed by EBV^+ve^ lymphomas, such as EBNA-1, are reduced in patients with endemic Burkitt’s lymphoma (BL), whereas responses to lytic or latency phase proteins not expressed by the tumors are preserved (18). Similarly, expression of T-cell “exhaustion” markers in Hodgkin lymphoma (HL) is associated with loss of function in EBV-specific CD8^+^ T cells (19). In patients with EBV^+ve^ nasopharyngeal carcinoma, the absolute frequency of LMP1-, LMP2-, and EBNA-1-specific CD8^+^ T cells is reduced in blood (20, 21), and these T cells appear to be functionally inactivated at the tumor site (22). These all suggest strong inhibition of T-cell responses specific for B lymphoma antigens. Tumor-induced T-cell exhaustion is well-characterized in many solid tumors [reviewed in Ref. (23, 24)], leading to similar patterns of tumor-specific T-cell dysfunction. Taking melanoma as an example, tumor infiltrating T cells appear to be rendered unresponsive locally in the tumor bed (25), and this can be associated with poor responses to adoptive immunotherapy (26), substantial expression of co-inhibitory molecules by tumor-specific T cells (27, 28), and transcriptional profiles consistent with exhaustion (29). Overall, these observations are consistent with tumor-associated T-cell dysfunction and tumor-specific tolerance.

## B Cells as Tolerogenic Antigen-Presenting Cells

Effector and memory differentiation of T cells results when co-stimulatory receptors (CD28, CD27, 41BB etc) are ligated by the high levels of ligands on activated antigen-presenting cells (APC) during cognate activation. But, in the absence of activation by pathogen- or danger-associated signals, APC provide insufficient signals for full T-cell activation and the outcome is peripheral T-cell tolerance. While DC are well recognized as potently tolerogenic cells in the steady-state (30, 31), reports extending back to the 1990s imply B cells are also tolerogenic (32–34). Some reports describe a direct role for B cells in peripheral deletion of naive CD8^+^ T cells, possibly by CD95-mediated effects (35). Others demonstrate contributions from both deletion and inactivation for naïve CD8^+^ T cells (36) and abortive proliferation appears to be a key requirement for tolerance induction (33, 36). More recent evidence suggests B cells may also inactivate memory CD4^+^ T cells (37). Moreover, we have shown that B cells expressing cognate antigens rapidly inactivate memory CD8^+^ T cells and CTL (38). Although “regulation” [e.g., through induction of regulatory T cells (39–41)] may be induced by B cells under certain conditions, T-cell intrinsic deletion and induction of unresponsiveness (anergy) are prominent when T cells interact with tolerogenic B cells. A subpopulation of IL-10-producing regulatory B cells (Breg) has also been described [reviewed in Ref. (42)]. These observations all suggest that B-cell lymphomas are potentially highly tolerogenic.

Preclinical B-lymphoma models in mice typically employ A20 lymphoma cells or transgenic Eμ-driven oncogenes such as c-Myc, and may express model neo-antigens like OVA in order to permit analysis of the impact on T cells (43). Although innate immune mechanisms impact on B-lymphoma in pre-clinical models, it is clear that inactivation of CD4^+^ and CD8^+^ T cells specific for tumor-expressed antigens also occurs (44–49). In models described to date, tolerance mechanisms are similar to those described for “tolerogenic B cells” with deletion and induction of unresponsiveness playing key roles (43–49). Whether the tumor cells themselves are tolerogenic or whether other APC presenting tumor-derived antigens are the proximal APC has been addressed. Whereas, host BM-derived APC appear to be required for CD4^+^ T cell tolerance in lymphoma models (44–46); it appears CD8^+^ T cells directly interact with antigen-expressing tumor cells (47–49). In fact, for CD8^+^ T cells, lymphoma cells appear to be the proximal APC for tolerance induction (49). Remarkably, OVA-specific CTL adoptively transferred into mice bearing OVA-expressing Eμ-myc lymphoma cells are rapidly deleted or rendered unresponsive (47, 49). This is remarkably similar to our own findings when CTL are transferred into mice where OVA is expressed within non-malignant B cells (38), which might suggest that this is an intrinsic outcome following the interaction between B cells and CTL.

## Immunological Synapse Structure and Function

**Figure 1:**
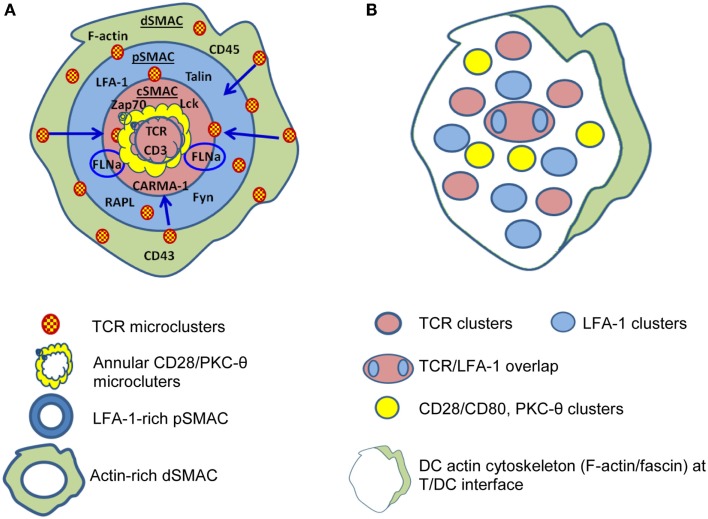
**Differences are apparent between immunological synapses formed by B cells and dendritic cells (DC)**. **(A)** B cells, B cell tumors, and lipid bilayers form classical “bulls-eye” immunological synapses. TCR-containing microclusters form in the dSMAC, contain Lck and ZAP70, protein kinase C (PKC-θ), LAT, SLP76 etc., and migrate centripetally through the LFA-1-rich pSMAC to the cSMAC. The cSMAC is segregated into a central CD3^hi^ region where CD3 accumulates and TCR is internalized to resulting in termination of TCR signaling and an outer CD3^lo^ region where the signaling molecules accumulate either in conjugation [annular CD28/PKC-θ conjugates and PKC-θ/filaminA (FLNa) clusters] or separately. (**B)** DC typically form “multifocal” synapses where TCR-containing clusters are segregated from CD28/PKC-θ containing clusters and no clear “ring” of LFA-1 is formed. TCR signaling stabilizes the multifocal structure, particularly the CD28/PKC-θ containing clusters. A prominent polarization of the DC cytoskeleton is often present at the periphery. Based on Ref. (51, 53, 55–57) **(A)**; (58–60) **(B)**.

A critical component of the interaction between T-cells and APC is formation of the immunological synapse (IS), defined as the contact area between a T cell and an APC presenting a peptide ligand. Various aspects of IS structure and function have been reviewed in detail (50–53), but a brief introduction will be provided here. Upon TCR ligation by pMHC, nanoclusters containing TCR begin to assemble around the initial site of APC/T-cell contact and these increase in size to form microclusters (MC) of TCR and associated molecules such as co-stimulatory receptors (e.g., CD28), tyrosine kinases (Lck and ZAP70), serine kinases [protein kinase C (PKC-θ)], and adaptor molecules (LAT, SLP76) (54). TCR MC begin to move toward the center of the ring of contact between the APC and T cell (Figure 1) where they aggregate to form a central supramolecular activation cluster (cSMAC) (55).

The cSMAC is surrounded by a ring of lymphocyte function associated antigen-1/intercellular adhesion molecule (LFA-1/ICAM) making up the peripheral SMAC (pSMAC) (55, 61, 62). After initial antigen recognition, the IS is stabilized by TCR-induced increases in LFA-1 affinity (63, 64). The pSMAC also contains LFA-1-associated proteins that regulate LFA-1 adhesion (55, 65) and LFA-1 here serves a crucial role in T-cell function by integrating internal cytoskeletal dynamics with the external environment (64, 66). This is mediated through, among other pathways, the actions of talin, an actin adapter protein, and RAPL, a Rap1 effector (55, 65) that modulate LFA-1 adhesion. In addition to its adhesive function, LFA-1 may be important by promoting pMHC/TCR localization to, and CD45 exclusion from, the cSMAC (67).

The pSMAC is surrounded by a more distal ring (dSMAC) containing membrane proteins with large ectodomains such as CD43 and CD45 (50, 68). The dSMAC appears to be the site of initial pMHC/TCR MC formation and, once formed, MC move centripetally through the pSMAC, facilitated by the concurrent centripetal movement of LFA-1/ICAM (69), to accumulate in the cSMAC (54, 70). Ca^++^ mobilization studies indicate TCR signaling commences with TCR MC formation in the dSMAC, and as MC move toward the cSMAC associations with ZAP70, Lck, LAT, and SLP76 are lost suggesting that by the time MC arrive at the cSMAC signaling capacity is lost (70). Additionally, MC in the cSMAC co-localize with markers of protein degradation and ubiquitinylation including Cbl-b (54, 71), a known inhibitor of TCR signaling. The cSMAC is also the site of TCR internalization for degradation (54, 72). Consistent with these observations, there is growing recognition that the cSMAC is a site for signal termination rather than stabilization of TCR signaling as originally thought [reviewed in Ref. (73)]. TCR signaling is initiated by the CD4 or CD8 co-receptors binding to the MHC molecules presenting cognate peptide, which activates the co-receptor-associated tyrosine kinase Lck. This in turn phosphorylates ITAM motifs within CD3-ζ. The tandem SH2-domains of ZAP-70 become engaged by the bi-phosphorylated ITAMs of CD3-ζ, and this then arranges ZAP-70 in a way that leads to phosphorylation of the transmembrane protein linker of activated T cells (LAT). Phosphorylated LAT, in turn, serves as a docking site to which a number of signaling proteins bind including SLP-76, which leads to signaling by the Ras-Erk pathway, and Ca^++^ flux [reviewed in Ref. (74)] and, ultimately, transcription of a range of gene products including those of immediate/early genes *c*-Fos, *c*-myc, *c*-jun, NF-AT, and NF-κB that ultimately lead to expression of IL-2, IL-2R, and other molecules that allow T cells to proliferate, differentiate, and exert effector function (75, 76).

An important point when considering the IS is that our understanding has been largely defined using *in vitro* models, some employing “artificial” APC, and hence, differences may exist between these and *in vivo* settings. For example, substantial differences in IS structure exist between different T-cell/APC combinations [reviewed in Ref. (53)]. Whereas classic “bulls-eye” IS are formed for T cell/B cell contacts (55, 77) and have been considered the “archetypal” IS, multifocal IS are characteristic of the interactions of DC with naive and activated CD4^+^ and CD8^+^ T cells, for example Ref. (58–60). Additionally, T-cell/DC conjugates develop in the absence of antigen (78) whereas T-cell/B-cell interactions do not (79). Interestingly, the antigen requirement for cytoskeletal rearrangement differs between T cells and DC. Naive CD4^+^ cytoskeletal polarization occurs during DC/T interactions in the absence of antigen, DC cytoskeletal polarization, and the formation of fully developed “multifocal” IS requires the presence of cognate pMHC (58, 80), suggesting that rearrangements in DC may be driven by the T cell.

## B-Lymphoma Induced Alterations in IS Formation

The “bulls-eye” IS formed between T cells and B cells (77) or B cell tumors (55) potentially favors damping of TCR signaling (73), but it is possible that altered IS formation by malignant B cells could contribute to perturbations of T-cell function. Indeed, altered IS formation between T cells and superantigen-pulsed malignant or healthy B cells has been observed in follicular lymphoma (FL), diffuse large B-cell lymphoma (DLBCL) (81), and in B-CLL (82, 83) as well as a mouse model of B-CLL (84). From these studies, it appears that several critical steps during and subsequent to IS formation are altered.

## Events Occurring within the cSMAC and Signaling Zone

Phosphorylation of ZAP-70 is crucial for signaling downstream of TCR. In the absence of ZAP-70 activity, formation of TCR/CD3ζ clusters and exclusion of CD43 from the cSMAC proceeds, but TCR-induced microtubule organizing center (MTOC) polarization and overall actin cytoskeletal changes and recruitment of signaling molecules such as PKC-θ and LAT to the T-cell/APC interface are impaired (85, 86). Interestingly, alterations in IS formation by CD4^+^ or CD8^+^ T cells from FL, DLBCL, and B-CLL (81–84) resemble those that occur in the absence of ZAP-70 activity (85, 86). For example, T cell/B cell conjugate formation rate is reduced and F-actin polymerization at the IS substantially impaired in CD4^+^ and CD8^+^ T cells isolated from tumor sites or the blood of leukemic-phase FL patients compared to healthy T cells or circulating T cells from non-leukemic phase FL (81). Disruptions in actin-based motility and cytoskeleton polarization have also been observed in acute myeloid leukemia (AML) (87).

Immunological synapse defects appear to be induced by tumor cells themselves, as impaired IS formation is induced in healthy allogeneic T lymphocytes by direct contact with FL, DLBCL, or B-CLL cells tumor cells (81, 82). Exposure to malignant B cells resulted in reduced recruitment of LFA-1 (particularly the high-affinity form), Lck, tyrosine-phosphorylated protein, Itk, filamin-A, and Rab27A to T-cell/APC contact sites (82), and these changes were apparent on re-culture with healthy B cells. Associated with this, functional alterations extended to reduced IL-2 production and CTL activity in T cells exposed to FL, DLBCL, or B-CLL cells (81, 82). Cell–cell contact was required and prevention of cell adhesion during the primary exposure to malignant B cells eliminated the effect (81, 82). These data suggest that interaction with malignant B cells could induce long-lived changes in T cells and, consistent with this, altered gene expression patterns have been detected in CD4^+^ and CD8^+^ T cells recovered from B-CLL patients and in tumor-infiltrating lymphocytes in FL (83, 88). Interestingly, the immunomodulatory drug lenalidomide, which shows effectiveness in B-lymphoma alone (89–91) or combined with Rituximab (92–94), could reinstate F-actin polymerization and signaling at the IS (81, 82).

## Co-Inhibitory Molecules within the IS

CTLA4 and PD-1 are co-inhibitory receptors that negatively regulate T-cell activation and act within the IS (Figure S1 in Supplementary Material). Their actions at the IS level may differ depending on the state of T-cell differentiation and the extent and site of ligand expression (95). If ligated during the initial activation of naive T cells by professional APC, co-inhibitory receptors can impart long-lived inhibitory effects on T-cell function (96, 97). While the effects of CTLA4 ligation may be most profound during the initial development of a T-cell response when priming is occurring in lymphoid tissues, PD-1 in addition to effects during priming, powerfully modulates effector responses in an apparently reversible manner (98). CTLA4 is normally stored in secretory granules but traffics to the cSMAC upon TCR activation (99) and accumulation at the cSMAC is required for its inhibitory function (100). CTLA4 has a higher affinity for CD80 and CD86 than CD28 and competes with CD28 resulting in termination of PKC-θ-mediated NF-κB signaling (100, 101), mainly through the prevention of the recruitment of the downstream scaffolding signaling protein, CARMA-1 to the cSMAC, which is critical for the NF-κB signaling pathway activation (102). Unlike CD28, CTLA-4 trafficking to the IS is directly related to the strength of TCR signaling, with higher levels occurring when TCR signal strength is greatest (99). CTLA4 has been reported to be strongly expressed by T cells in HL (103), and may contribute to damping of T-cell function. In line with this, administration of CTLA4-blocking antibodies such as Ipilimumab appears to have antitumor activity in DLBCL and FL patients (104) and following HSCT for HL and mantle cell lymphoma (105). Although testing of anti-CTLA4 appears limited, it is currently being tested in combination with anti-PD-1. Interestingly, polymorphisms of *CTLA4* have been associated with increased susceptibility to NHL in some populations (106).

PD-1 is expressed by antigen-stimulated T cells and, in chronic viral infection, contributes to T-cell “exhaustion” (98, 107), where blockade can reinvigorate T-cell function, allowing expansion and production of effector cytokines (108, 109). Other co-inhibitory molecules appear to work in a similar way and PD-1 can act in conjunction with other co-inhibitory receptors (23). Expression of co-inhibitory receptor ligands such as PD-L1 by tumors is associated with poor prognosis (110–113). For example, PD-L1 is over-expressed in DLBCL and may contribute to poor outcomes (114, 115). In mantle cell lymphoma, PD-L1 expression inhibits T-cell proliferation and T-cell lytic activity (116). Similar results have been reported in a murine AML model (117). Engagement of PD-1 concurrently with TCR ligation impairs TCR-induced phosphorylation of CD3ζ, ZAP-70, and PKC-θ (118). PD-1 expressed on the surface of effector T cells is recruited to TCR MC upon their formation and is translocated to the cSMAC within the MC (119) and the higher the ligand availability, the more localization of PD-1 at the IS (120). During this process, SHP-2 is recruited to the immunoreceptor tyrosine-based switch motif (ITSM) of PD-1, which in turn causes dephosphorylation of TCR proximal signaling molecules within MC (119) impairing TCR-induced “stop” signals required for T-cell activation (121). Blockade of PD-1 ligation partially restores IS formation between healthy T cells and CLL cells (122). Blockade of co-inhibitory receptor/ligand interactions through a PD-1 antibody promotes T-cell function and immune-clearance of solid tumors (123, 124), and early indications suggest a similar effect in FL (125) and HSCT for relapsed or refractory DLBCL (126). The use of anti-PD-1 antibody has been extended and combined with the anti-CD20 Ab Rituximab in relapsed FL (127). Generally, the use of PD-1 blockage has shown promising outcomes in the case of lymphomas (128).

## Events Outside the cSMAC

**Figure 2:**
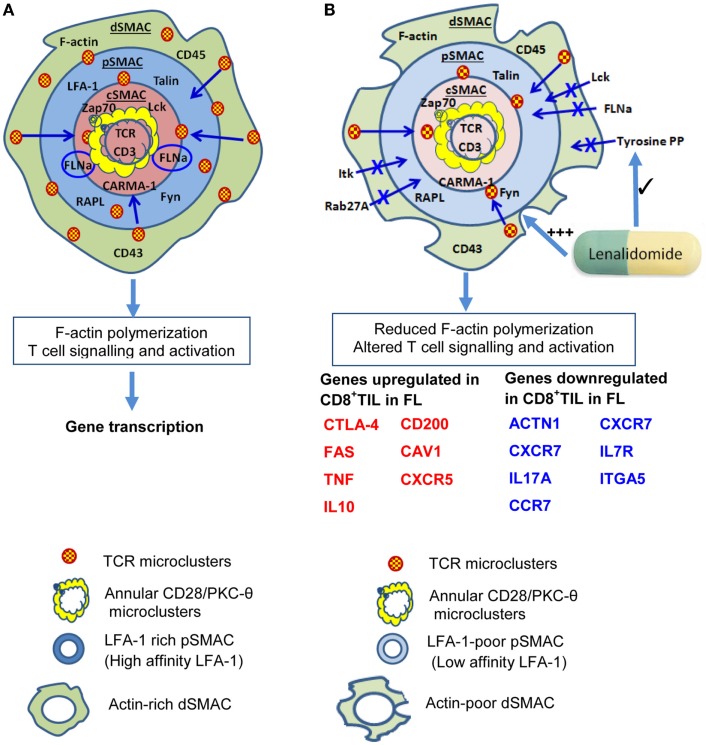
**Impaired immunological synapse formation at the T-cell/B-lymphoma interface**. **(A)** The stylized, classical “bulls-eye” IS as depicted in Figure 1. (**B)** During interaction with B lymphoma cells, IS formation is altered and a reduction in actin cytoskeletal remodeling is evident in the dSMAC. This likely leads to reduced formation and centripetal migration of TCR/CD28 microclusters. As a result of altered cytoskeletal remodeling, dSMAC formation is incomplete, and Lck, Itk, filaminA (FLNa), tyrosine-phosphorylated protein, and Rab27A recruitment to the IS are also reduced. Consequently, the pSMAC contains reduced amounts of LFA-1 and this is primarily the low affinity form which destabilizes T-cell: B-lymphoma conjugate formation. This leads to reduced tyrosine phosphorylation and TCR signaling leading to altered downstream gene transcription. Reduced TCR signaling could also perpetuate impaired IS formation through reduced ZAP-70 signaling. Increased transcription of genes encoding some molecules that inhibit T-cell activity or regulate motility or division (e.g., CTLA-4,FAS, TNF, IL10, CD200) occurs in CD8^+^ tumor-infiltrating lymphocytes (CD8^+^ TIL) as well as reduced transcription of some molecules that contribute to efficient IS formation [e.g., actinin-1 (ACTN1)], IL7R, CCR7, ITGA5. Lenalidomide acts to restore F-actin polymerization, rho-GTPase signaling, recruitment of tyrosine-phosphorylated protein (tyrosine PP), and also improves conjugate formation between T cells and B lymphoma cells. Based on Ref. (51, 53, 55–57) **(A)**; (81, 88) **(B)**.

Malignant B cell-induced alterations in the IS extend beyond the cSMAC (Figure 2). Stabilization of pSMAC LFA-1/ICAM-1 interactions are impaired in T cells from FL and B-CLL patients (81, 82). Alterations in Rho-GTPase signaling that likely underlie these IS alterations (83, 129) also appear to perturb LFA-1 mediated migration. Perhaps, more pertinent for the topic under consideration, effective LFA-1/ICAM interactions are required for memory T-cell differentiation. In the absence of ICAM-1-mediated stabilization of the IS, long-lived T-cell/DC conjugates are reduced in frequency (130). While this has little effect on T-cell activation, proliferation, and cytotoxicity, a key outcome is failure of activated T cells to develop effective memory populations and clonal deletion of activated T cells (130). It is plausible if perturbed LFA-1/ICAM-1 interactions led to similar outcomes in human T-cells, this could underlie the reduction in frequency and loss of responsiveness of EBV-reactive T cells in EBV^+ve^ lymphoma.

## Future Directions

Understanding the mechanistic origins of IS alterations in lymphoma is an area that could significantly advance therapy. Transcriptional profiling has provided insight into pathways through which altered IS structure and function are potentially established and downstream effects mediated (83, 88). Several areas of investigation are likely to be fruitful, but fundamental questions remain. We have principally discussed the role of lymphoma cells as APC for T-cell activation, but clearance of B-cell malignancies also requires CTL recognition of malignant cells. This is an understudied area, and dissecting the role of malignant B cells as “activating APC” for CTL will require further sophisticated studies.

It is intriguing, however, to consider whether antigen-specific tolerance mechanisms contribute and whether this could be a cause or consequence of altered IS function. An outstanding question is whether functional alterations observed in T cells is a global effect or the consequence of cognate tumor interactions that affects only tumor-antigen specific T cells. For instance, does impaired IS formation occur during the primary interactions of T cells with malignant B cells in a way that programs subsequent outcomes for those T cells? Impaired priming of T cells to a model antigen in a mouse model of B-CLL (84) suggests global effects, and clinical (82) and mouse studies (38, 47, 49) suggest tumor burden is an underlying determinant of the effect. Rituximab treatment restores immune responsiveness in FL in keeping with a suggestion that reduction in tumor burden may reduce the effect on T-cell dysfunction (131). On the other hand, some mouse studies indicate that T-cell dysfunction is restricted specifically to T-cells that display specificity for lymphoma cells (49), indicating tumor antigen-specificity of the effect, and T-cell dysfunction in B-lymphoma shows some evidence of specificity for tumor-derived antigens (18–22). Many of the IS alterations reported for T cells from lymphoma patients could be caused by proximal defects in TCR signaling (38, 132) found in tolerant T cells. Tolerant T-cells demonstrate impaired translocation of ZAP70, LAT, and phospholipase C γ1, into the IS and IS formation (133–137). Further investigation may reveal whether antigen dose/affinity effects on ZAP-70 signaling and TCR damping molecule recruitment (71, 138, 139) or modulation of lipid rafts (140–142) underlie some of the effects observed. *In vitro* visualization of the defects of the IS and testing the capacity of pharmacological agents such as lenalidomide (89–91) or co-inhibitory receptor blockade to modulate this, using live cell and confocal microscopy, might be a promising transitional step for a more advanced understanding.

## Summary

It is apparent many processes are perturbed at the IS in B-lymphoma. Several of these processes may act in concert to inhibit generation of effective T-cell responses to malignant B cells. Alternatively, a small number of processes with widespread influences may underlie the changes observed. Further characterization is required to determine whether “defects” observed are “downstream” of other tumor effects or whether the alteration in IS function described is the primary cause of failure of effective T-cell immunosurveillance. This is an area that could provide useful insights for the development of more effective therapies for B-cell and other malignancies.

## Author Contributions

MR, JW, and RS wrote the manuscript.

## Conflict of Interest Statement

The authors declare that the research was conducted in the absence of any commercial or financial relationships that could be construed as a potential conflict of interest.

## Supplementary Material

The Supplementary Material for this article can be found online at http://journal.frontiersin.org/article/10.3389/fimmu.2015.00258/abstract
