# Supplementary figures and images for: Impaired T-Cell Function in B-Cell Lymphoma: A Direct Consequence of Events at the Immunological Synapse?
Source: Front Immunol. 2015 Jun 2;6:258. doi: 10.3389/fimmu.2015.00258 (PMC4451642; doi:10.3389/fimmu.2015.00258)

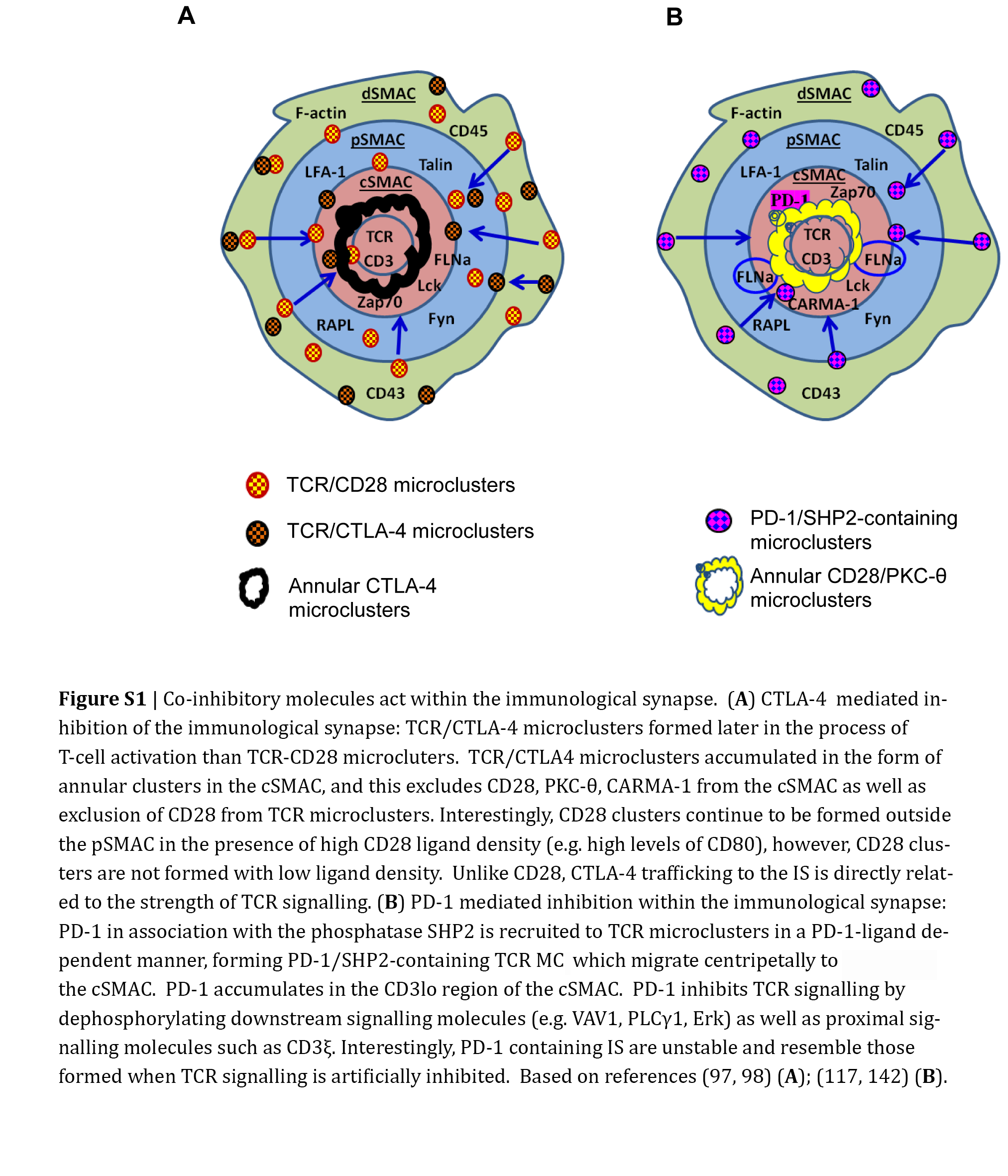

Supplement: Supplementary file 1 [file image_1.tif]
